# Stress and coping among dual caregivers in China: a grounded theory study of intergenerational caregiving

**DOI:** 10.3389/ijph.2026.1610111

**Published:** 2026-09-23

**Authors:** Han Hu

**Affiliations:** School of Public Policy and Administration, Xi’an Jiaotong University, Xi’an, China

**Keywords:** caregiver stress, coping strategies, dual caregivers, grounded theory, intergenerational caregiving

## Abstract

**Objectives:**

To examine how adults who simultaneously care for older adults and children aged 0–6 years experience competing demands, mobilize resources, and negotiate caregiving decisions in two Chinese cities.

**Methods:**

We conducted a qualitative grounded theory study with 23 dual caregivers in Shanghai and Xi’an. Participants were recruited from an online questionnaire pool and interviewed using a semi-structured guide. Interview transcripts were analyzed through constant comparison and iterative coding.

**Results:**

The analysis generated a feedback process linking continuous and competing care demands, secondary strains, conditional resources, and negotiated outcomes. Overlapping, urgent, and difficult-to-substitute tasks extended strain into work, finances, psychological wellbeing, and family relationships. Family help, formal and market services, and self-regulation widened caregivers’ room for action only when they were accessible, reliable, affordable, and matched to specific tasks. Caregivers formed four changeable strategy configurations and made varied adjustments to work, further childbearing, and future planning.

**Conclusion:**

Dual caregiving is a dynamic process in which competing demands, resource allocation, and strategy adjustment reinforce one another. Continuous, affordable, and time-matched support may strengthen families’ capacity to sustain care.

## Introduction

Adults who simultaneously support older relatives and dependent children are commonly described as sandwich-generation or dual caregivers [1]. National evidence from the United States indicates that, compared with caregivers of older adults alone, dual caregivers more often report role overload and financial and emotional strain [2]. The underlying pressures are not confined to one society. Population ageing, lower fertility, and changes in family structure are widening the gap between care needs and families’ capacity to meet them in many settings [3, 4]. Their specific expression nevertheless depends on the interaction of household structure, labor-market conditions, service availability, and cultural expectations.

Dual caregiving is not simply the sum of two independent roles. Young children require continuous supervision, transport, and educational support, whereas illness, functional limitation, or medical appointments among older relatives may interrupt established routines without warning. Pearlin’s stress process model provides a useful starting point by distinguishing primary stressors arising directly from care demands from secondary strains in employment, finances, identity, and relationships, while recognizing social and personal resources as potential mediators [5]. Subsequent research shows that care intensity, employment conditions, caregivers’ health, and available support jointly shape burden and wellbeing [6–8]. What remains less clear is how stress spreads across domains when eldercare and childcare overlap, and why resources protect some options while leaving other costs unresolved.

Sociocultural models further suggest that cultural values and family relations shape how caregivers interpret obligations and seek help [9, 10]. Studies in China show that family duty, competition between eldercare and childcare, and intergenerational living arrangements create particular contexts for allocating care [11–13]. Research in other settings has identified task delegation, help-seeking, time management, self-soothing, distancing, and goal adjustment as coping responses [14–17]. However, these actions are often examined separately. Less attention has been paid to how caregivers combine generational priorities, child-rearing expectations, employment decisions, and intentions for further childbearing under different configurations of family, service, and personal resources.

We therefore conceptualized the stress process as a resource-allocation process with feedback. Direct care demands generate immediate strain; secondary work and financial pressures may then reduce the time and money available for subsequent care; caregivers respond by reorganizing tasks, purchasing services, or adjusting goals. The same response may protect daily family functioning while transferring costs to health, employment, or future care capacity. This study asked: (1) What pressures arise when care for older adults and children aged 0–6 years overlaps? (2) How do caregivers mobilize family, formal, market, and personal resources? (3) How do different resource conditions correspond to strategies involving generational priorities, child-rearing expectations, work, and further childbearing? We used interview data to generate categories and subsequently used the stress process model to organize their relationships into an empirically bounded process explanation.

## Methods

### Study design and sampling

This qualitative study followed the grounded theory procedures described by Strauss and Corbin [18] and was reported with reference to the Consolidated Criteria for Reporting Qualitative Research [19]. An online questionnaire conducted through communities in Shanghai Municipality and Shaanxi Province between March and June 2024 yielded 2,064 valid responses. It collected structured information on caregivers, care recipients, care arrangements, support, employment, and six author-designed burden domains. At the end, respondents could indicate willingness to be interviewed and voluntarily provide a telephone number. Questionnaire information was used only to construct the recruitment pool, support purposive sampling, and describe participants; it was neither analyzed as an outcome dataset nor integrated into interview coding. Academic and policy materials were used to frame the question and interpret findings, not as coded empirical data. Table 1 distinguishes the role of each source.

**TABLE 1:** Distinct analytical roles of the questionnaire, interviews, and background materials (Shanghai and Xi’an, China. 2024).

Source	Content	Function	Analytical boundary
Semi-structured interviews	March–August 2024; 10 face-to-face interviews in Xi’an and 13 telephone interviews; 50–90 min; verbatim transcription	Generated initial concepts, focused categories, properties, empirical dimensions, and the process model	All empirical findings and quotations in this article derive from the 23 interviews
Online questionnaire	March–June 2024; 2,064 valid responses; care arrangements, family support, employment, and six author-designed burden domains; optional follow-up contact	Constructed the recruitment pool, supported maximum-variation purposive sampling, and described participants	Not a validated burden scale; no outcome analysis; not coded with interview transcripts
Academic and policy background materials	Peer-reviewed literature and policy materials related to caregiving	Framed the research question and supported discussion of the interview interpretation	Not empirical data for this study and not included in coding

Shanghai and Xi’an were selected to obtain experiences from contrasting urban resource and service environments, not to represent China statistically. Respondents from the Shaanxi recruitment pool who entered the interview sample lived in Xi’an. Interview eligibility required participants to be adults who simultaneously provided unpaid care to at least one child aged 0–6 years and at least one older adult aged 60 years or above, had cared for the older adult for at least six consecutive months, could understand the questions, and could provide informed consent. The first stage used maximum-variation purposive sampling [20] across residence, income, gender, employment, household composition, and response patterns in the six burden domains. The domains were not combined into a validated score, and no high-burden threshold was used. As analysis progressed, developing categories guided theoretical sampling among respondents who had volunteered for follow-up.

The final sample comprised 23 caregivers, including 17 women and six men, aged 32–72 years (mean 44.5 years). Fifteen were employed; 15 lived in Xi’an and eight in Shanghai. Variation supported comparisons across resource conditions but did not create statistical subgroups or proportional representation. Participant characteristics are presented in Table 2 and Supplementary File 1.

**TABLE 2:** Characteristics of the 23 interview participants (Shanghai and Xi’an, China. 2024).

Characteristic	n	%
Sex		
Women	17	73.9
Men	6	26.1
Age, years		
30–39	12	52.2
40–49	3	13.0
50 or older	8	34.8
Education		
College or below	13	56.5
Bachelor’s degree or above	10	43.5
Health status		
Healthy	16	69.6
Minor illness	6	26.1
Serious chronic illness	1	4.3
Employment		
Employed	15	65.2
Not employed	8	34.8
Children receiving care		
1	16	69.6
2 or more	7	30.4
Older adults receiving care		
1	14	60.9
2 or more	8	34.8
Not recorded	1	4.3

Maximum variation was used for analytical comparison, not to represent demographic strata. We examined whether similar demands produced different responses as employment flexibility, available relatives, the number of care recipients, or access to paid help varied. Contrasting accounts were retained, including those combining fatigue with a sense of meaning and those maintaining intensive child-rearing while prioritizing older adults. These comparisons informed later sampling and refinement of category boundaries.

### Data collection

The semi-structured guide covered household composition and finances, care tasks and intensity, conflict and prioritization, effects on work and further childbearing, available coping resources, and desired support. Following pilot testing, ten face-to-face interviews were conducted in Xi’an from March to May 2024 and 13 telephone interviews from June to August 2024. All interviews lasted 50–90 min, were audio-recorded with permission, and were transcribed verbatim. Both modes used the same guide and probing logic. The study was not designed to compare duration or disclosure by mode; the possible influence of mode is addressed as a limitation.

Interviewers began by asking participants to describe a typical day and the tasks performed for each care recipient. They then probed pressure, conflict, sources of help, time spent on tasks, responses when needs overlapped, and the reasons for prioritizing one demand. Separate 1–10 ratings of the intensity of older-adult care and childcare were used only to prompt explanations of tasks and experiences, not as quantitative outcomes. Later interviews compared child-first and older-adult-first decisions and intensive and basic child-rearing expectations.

Two trained researchers conducted the one-to-one interviews. Two team members checked transcripts against the recordings, and a third reviewed discrepancies. Data collection and preliminary analysis proceeded concurrently. After interview 20, no new higher-order category was identified. Three additional interviews were conducted to test category boundaries and confirm theoretical sufficiency, consistent with evidence that focused qualitative studies may reach code or meaning saturation within this range while requiring explicit assessment of category depth [21].

### Data analysis

Analysis used constant comparison and open, axial, and selective coding [18]. Three researchers first coded transcripts line by line to develop initial concepts. They then compared similarities and differences, grouped concepts into focused categories, specified properties and dimensions, and examined relationships among conditions, actions, and perceived consequences. Selective coding integrated the core process. Successive analytic matrices recorded category definitions, supporting quotations, negative cases, and changes across coding rounds. Table 3 preserves the progression from initial concepts to categories, properties, empirical dimensions, and theoretical modules.

**TABLE 3:** Initial concepts, focused categories, properties, and empirical dimensions generated from interview analysis (Shanghai and Xi’an, China. 2024).

Theoretical module	Focused category	Property	Empirical dimension	Illustrative initial concepts
Direct care demands	Continuous and competing care demands	Physical load	Intensity and bodily limits	Heavy work; exhaustion; cannot become ill
Direct care demands	Continuous and competing care demands	Temporal rhythm	Continuous, interrupted, and sudden	Constant supervision; disrupted plans; compressed rest
Direct care demands	Continuous and competing care demands	Task complexity	Routine and specialized tasks	Feeding; transport; medication; hospital visits; bedridden care
Direct care demands	Continuous and competing care demands	Demand competition	Child-first, older-adult-first, or simultaneous coordination	Needs coincide; urgency is difficult to predict
Secondary strains	Work and financial strain	Work-time conflict	More or less negotiable	Leave; overtime; school pick-up and medical-visit conflicts
Secondary strains	Work and financial strain	Employment continuity	Stable or impaired	Reduced hours; stalled advancement; exit from paid work
Secondary strains	Work and financial strain	Financial capacity	Absorbable or constrained	Mortgage; tuition; medical costs; insufficient savings
Secondary strains	Psychological and self strain	Fear of uncertainty	Lower or higher	Concern about disability, emergencies, and future care
Secondary strains	Psychological and self strain	Self-neglect and role restriction	Manageable or severe	No personal time; cannot become ill; contracted goals
Secondary strains	Psychological and self strain	Social participation	Maintained or reduced	Declining invitations; reduced leisure; minimal social activity
Secondary strains	Family relationship strain	Task allocation	Clear or ambiguous	Spousal or sibling sharing; responsibility avoidance
Secondary strains	Family relationship strain	Relationship quality	Cooperation or conflict	Help and friction coexist; repeated disagreement
Secondary strains	Family relationship strain	Responsibility identity	Meaning and tension	Voluntary care; filial duty; guilt and fatigue
Conditional resources	Family support	Availability of helpers	More or fewer	Spouses; siblings; older relatives; adult children
Conditional resources	Family support	Reliability	Continuous or temporary	Stable division; temporary substitution; emergency availability
Conditional resources	Family support	Negotiation cost	Lower or higher	Whether seeking help triggers conflict
Conditional resources	Formal and market services	Accessibility	Available or unavailable	Community services; domestic help; childcare; home care
Conditional resources	Formal and market services	Affordability	Affordable or unaffordable	Service fees; household ability to pay
Conditional resources	Formal and market services	Trust and time match	Acceptable or uncertain	Service quality; trust; alignment with care schedules
Conditional resources	Self-regulation	Consumption and reserves	Reduced spending or protected resources	Lower consumption; emergency savings
Conditional resources	Self-regulation	Time and work adjustment	Efficiency, proximity, and stability	Precise scheduling; avoiding overtime; work near home
Conditional resources	Self-regulation	Goal and expectation adjustment	Maintained, postponed, or lowered	Intensive or basic child-rearing; work and fertility adjustment
Conditional resources	Self-regulation	Self-maintenance	Physical and psychological regulation	Exercise; brief rest; acceptance; meaning
Negotiated outcomes	Four strategy configurations	Generational priority and child-rearing expectation	Four illustrative combinations	Child-first/intensive; child-first/basic; older-adult-first/intensive; balanced/basic
Negotiated outcomes	Work responses	Employment adjustment	Protective adjustment or involuntary contraction	Efficiency; stability; reduced hours; stalled promotion; exit
Negotiated outcomes	Further childbearing	Fertility plans	Postponement or no further birth	Concern about capacity or reduced child-rearing quality
Negotiated outcomes	Perceived future control	Resources and expectations	Relatively controllable or constrained	Education and eldercare concerns; firefighting; no long-term plan

Analytic memos documented how each category developed. They asked when pressure intensified, which resources altered the available response, and what costs followed. During axial coding, cases were compared across employment situations, household arrangements, and resource conditions. Accounts that did not follow the dominant pattern were retained to refine category boundaries. These comparisons showed that generational priority and child-rearing expectations varied independently, which supported the four strategy configurations.

Coding was interpretive and iterative. The researchers discussed differences until consensus rather than treating agreement as a quantitative reliability exercise. Open and axial coding were predominantly inductive. Pearlin’s stress process model was not imposed as an *a priori* coding framework; it was introduced after the empirical categories had stabilized to organize relationships among direct care demands, secondary strains, conditional resources, and negotiated outcomes [5]. Neither questionnaire responses nor background materials underwent first- or second-order coding.

### Ethics

The questionnaire and interview components were approved by the institutional ethics committee (approval number 2016–416; June 30, 2016). The questionnaire landing page explained the study purpose, anonymity, voluntary participation, and the right to withdraw; continuing to the questionnaire indicated electronic informed consent under the approved protocol. Providing contact information for a follow-up interview was optional. Interview participants provided written informed consent before participation. Contact details were used only for recruitment, and analytic data were de-identified. The study followed the Declaration of Helsinki. The institution name is withheld from the blinded manuscript and will be provided in the submission system.

## Results

### An integrated process of stress, resources, and negotiated trade-offs

The analysis generated four interrelated modules: (1) continuous and competing care demands; (2) secondary strains affecting work, finances, psychological wellbeing, and family relationships; (3) conditional resources from family, formal and market services, and self-regulation; and (4) negotiated outcomes, including strategy configurations, work and childbearing adjustments, and perceived control over the future. Figure 1 presents their process relationships, and Table 3 shows the analytic chain from initial concepts to theoretical modules.

**FIGURE 1:**
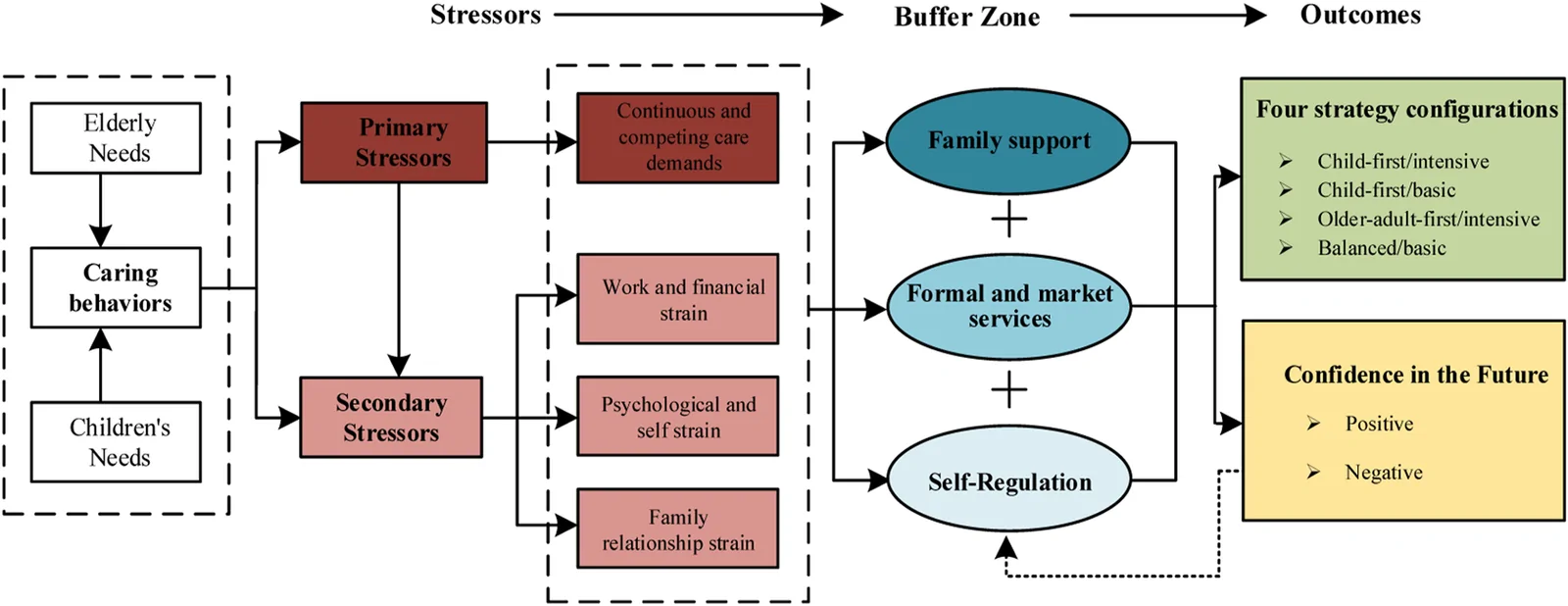
Process model linking competing care demands, stress proliferation, conditional resources, and negotiated outcomes among dual caregivers (Shanghai and Xi’an, China. 2024).

The modules were recursive rather than linear. Direct care demands created immediate strain, while work and financial strain could reduce the time and money available for later care. Support could interrupt this process, but its effects depended on family relationships, service access, and caregivers’ willingness or ability to seek help. Negotiated trade-offs could stabilize daily arrangements while generating new concerns about employment, children’s opportunities, older adults’ future dependence, and household resilience.

### Continuous and competing care demands

Young children required feeding, transport, supervision, companionship, medical visits, and educational support. Older adults could require household help, medication management, hospital accompaniment, mobility assistance, and emotional support. Although the tasks followed different rhythms, they often competed for the same time, attention, and physical capacity. A 55-year-old woman caring for a three-year-old grandchild explained:

“I think looking after a child is even more tiring than caring for an older person. You have to watch the child constantly and follow them everywhere.”

A 35-year-old woman described how pressure accumulated through small but non-deferrable tasks: *“I get up at around seven every morning and take my eight-month-old baby to my mother-in-law’s home. Then I have breakfast; when I return, she goes to eat. We alternate like this throughout the day.”* Functional dependence among older adults created a different form of intensity. A 46-year-old woman caring for two children and her father said:

“The hardest time is when an older person becomes completely bedridden and cannot care for themselves. You have to stay beside them constantly.”

“Continuous and competing care demands” therefore refers to a state of sustained availability and coordination, not literal uninterrupted care for every participant. Some considered child supervision more exhausting; others regarded loss of mobility among older adults as the greater pressure. The central issue was the coincidence, urgency, and limited substitutability of needs rather than one generation consistently demanding more care.

### Secondary strains across work, finances, self, and relationships

Care demands extended into paid work and household finances. Employed participants arranged work around school pick-ups, medical appointments, and eldercare emergencies. A 41-year-old divorced woman explained: “If I cannot stand out at work, the only thing I can do is stay longer. I leave early and return late every day, so during the working week I have almost no time to care for my parents.” Housing, tuition, childcare, and possible medical expenditure made future risks difficult to absorb. One participant said:

“I pay the mortgage and my child's tuition every month. The financial burden is immense. Many ordinary families have no savings now, which makes life even harder to manage.”

Psychological strain appeared as worry, irritability, guilt, and loss of time for self-care. An older woman summarized this constraint: ‘I spend every day looking after other people and have no time to care for myself. For me, getting ill is simply not an option.’ When responsibility was unclear or tasks were unequally distributed, negotiation itself became a secondary strain. One participant observed that *“the more family members there are, the more likely disagreements become.”* Care also narrowed social roles as participants reduced leisure, declined invitations, or avoided long-term commitments. At the same time, some valued closeness to children or older relatives and rejected an entirely burden-centered account.

### Layered but unequal conditional resources

Participants combined three types of resources. First, spouses, siblings, older relatives, and adult children shared tasks, costs, or emotional support when available. Second, some households considered paid domestic help, community services, or public assistance, but access, affordability, and confidence in service quality varied. One participant combined self-reliance with conditional openness to formal help:

‘I do not expect too much from any person or organization; I mainly rely on myself. But if I genuinely could not manage and the government or society happened to provide such a service, it would save me enormous trouble.'

Third, caregivers adjusted expectations and expenditure, protected small amounts of personal time, exercised, saved for emergencies, or reframed care as responsibility. For some, family obligation gave care meaning:

“I do not expect anything in return for caring for my grandchild. I do it entirely willingly. Respecting older people and caring for children is a Chinese tradition; it is my responsibility.”

Meaning and fatigue nevertheless coexisted.

The resources substituted for one another. When formal services were unavailable or distrusted, households relied more heavily on spouses and siblings. When relatives could not help, caregivers used savings or purchased services. When neither was sufficient, participants tightened schedules, reduced expenditure, or lowered achievement expectations. Substitution kept current care from breaking down, but it could transfer unresolved costs to caregivers’ health, employment, or future plans. Resources therefore buffered strain only when they were accessible, reliable, affordable, and matched to the relevant task and time.

### Four strategy configurations and uncertain futures

When time, money, and available helpers were insufficient, caregivers negotiated along two axes: generational priority when the needs of older adults and children conflicted, and the intensity of child-rearing investment that could be sustained. Their intersection produced four illustrative strategy configurations (Figure 2). These configurations are analytical combinations, not fixed personality types or estimates of prevalence.

**FIGURE 2:**
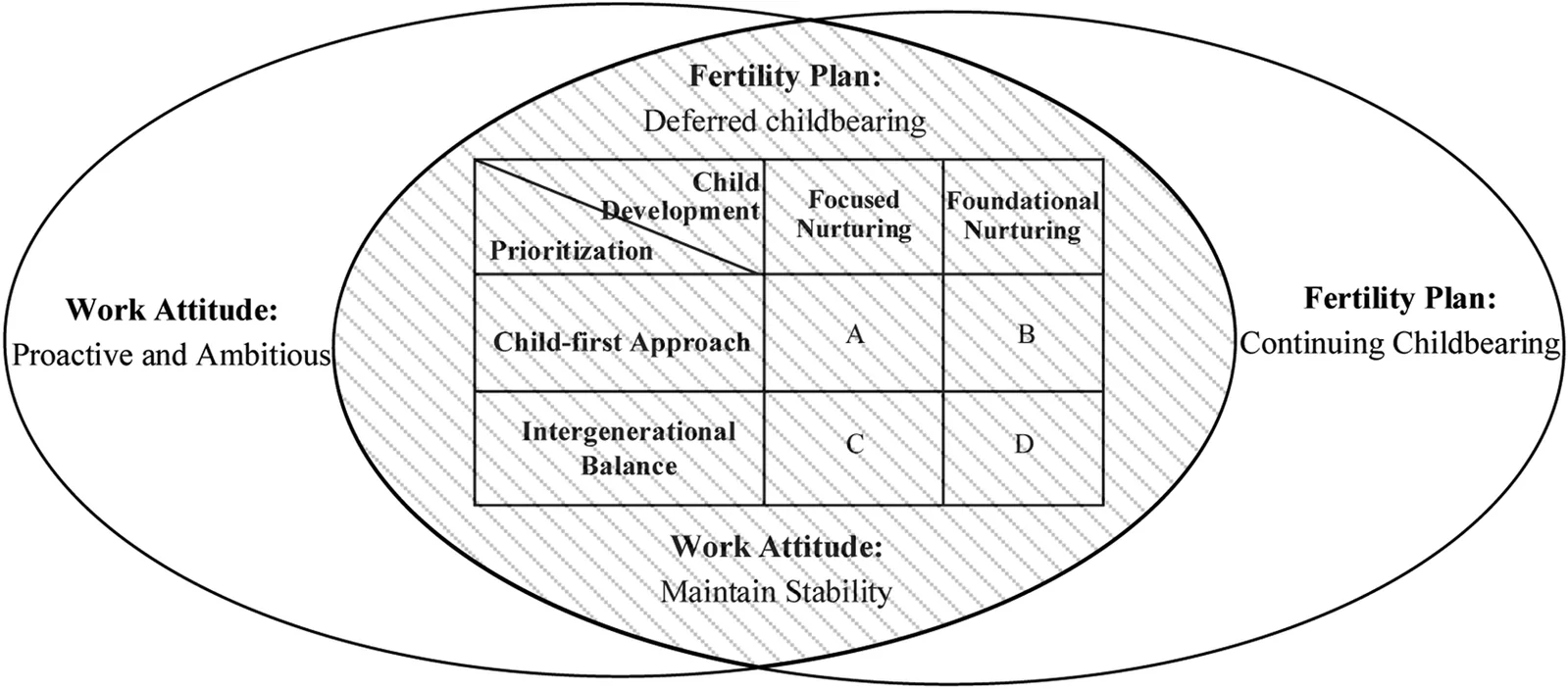
Four illustrative strategy configurations combining generational priority and child-rearing expectations among dual caregivers (Shanghai and Xi’an, China. 2024).

Configuration A, child-first with intensive investment, was illustrated by a comparatively well-resourced Shanghai household. Paid childcare and extended-family help supported extracurricular education and substantial parental involvement while older-adult care was shared.

Configuration B, child-first with basic provision, was illustrated by a lower-resource Xi’an household in which a woman caring for two children and older relatives prioritized children’s immediate food, schooling, and safety, reduced extracurricular spending, and relied on her husband’s siblings for some eldercare. The same generational priority thus corresponded to different child-rearing goals and negotiation costs.

Configuration C, older-adult-first while maintaining intensive child-rearing, was illustrated by a Shanghai father who valued children’s diet, psychological wellbeing, early education, and interests but prioritized his mother when both generations’ needs could not be met: *“With older people, every day together is 1 day less, and every day is precious. My child still has a long road ahead.”*

Configuration D, balanced generations with basic provision, was illustrated by a single mother in Xi’an who set no fixed generational priority and reduced extracurricular expectations to preserve room for coordination: “*We are a very ordinary family. I do not insist that my child learn an instrument or a special skill. I am raising my child as I was raised, in a relaxed and free way.”*

The configurations show that the same generational priority can coexist with different levels of child-rearing investment, while similar educational expectations can accompany different priorities. They are temporary couplings among the urgency of needs, family norms, educational goals, and substitute resources. Participants could move between configurations as older adults’ functioning, children’s needs, employment, or available help changed.

Neither configuration membership nor generational priority determined a single employment or fertility response. Employment adjustments depended on job flexibility, income needs, household task-sharing, and whether substitute care could cover specific hours. Further-childbearing decisions were shaped by perceived future capacity as well as current care demands. Work and fertility were therefore related to the broader resource-allocation process but remained analytically distinct negotiated outcomes, with different combinations of stability, contraction, postponement, and uncertainty across participants.

Some participants connected their present care situation to decisions about further childbearing. One said: *“I have ruled out having a second child. At 40, I am already overwhelmed. Raising another child from infancy while caring for ageing parents is beyond what I can manage.”* These accounts indicate perceived constraints rather than a uniform fertility response. Work responses also varied. Some participants improved efficiency or chose employment close to home, whereas others reduced hours, experienced stalled advancement, or left paid work. One explained: *“We plan every minute precisely and never volunteer for overtime, so that we can collect the child from school on time.”*

Even when current arrangements were relatively stable, perceived control over the future was eroded by possible functional decline among older adults, educational costs, and health emergencies. One participant said: *“When I think that my parents might 1 day be completely bedridden, I am terrified. I cannot imagine how I would cope.” Another said, “Our family could not withstand a serious illness or accident.”* A third described daily life as “firefighting” rather than long-term planning. Perceived future control therefore connected current strategies to the next cycle of stress: short-term adjustments maintained care but could limit savings, career development, and preparation for future needs.

## Discussion

### Main findings and theoretical contribution

This study conceptualizes dual caregiving as an interconnected process of demand competition, stress proliferation, resource modulation, and strategic negotiation. Continuous and competing needs first produced time scarcity and physical fatigue, then extended into work, finances, psychological wellbeing, and relationships. Family task-sharing, formal and market services, and self-regulation altered the options available, but the four strategy configurations were not endpoints. They were provisional arrangements through which households maintained continuity of care under specific resource conditions.

The findings refine the stress process perspective in three ways. First, primary stressors cannot be understood only through total care volume. Temporal overlap, urgency, and task substitutability jointly shaped perceived competition. Second, secondary strains operated as feedback: employment reduction and financial strain were consequences of care but also reduced later income, time, and room for action. Existing evidence on formal and informal support, family task-sharing, and caregiver wellbeing supports treating resources relationally rather than as uniformly protective [22, 23], while longitudinal studies of financial strain, employment, and care intensity provide external support for the possibility of cumulative feedback [24, 25]. Third, negotiated outcomes had a dual character. They protected basic care in the short term but could transfer risk to health, careers, children’s opportunities, or future care capacity.

This interpretation also shifts attention from the amount of support to task-resource fit. A relative may provide emotional reassurance but be unavailable for hospital accompaniment; money can purchase help only where an acceptable service exists at the required time; and flexible work protects employment only when tasks and supervisors permit its use. Resources were therefore conditional in both practical and relational terms. Their value depended on accessibility, reliability, affordability, timing, and the negotiation costs attached to seeking help. This explains why nominally similar households could retain very different rooms for action.

### Shared pressures and the Chinese context

Fatigue, time conflict, financial strain, work-family conflict, and family negotiation have been reported across societies and should not be presented as uniquely Chinese [26–29]. Service use may moderate some adverse consequences, although effects depend on access, care needs, and family relationships [30]. The Chinese context in these interviews lay mainly in how participants interpreted shared pressures through intergenerational reciprocity, filial responsibility, and educational expectations, and in which substitutes were available where formal care was uneven. Child-first, older-adult-first, and balanced arrangements should therefore be understood as contextual responses rather than stable cultural types.

Gendered task allocation and multiple-role structures also shape caregiving burden [31, 32]. Our predominantly female sample reflects, but cannot quantify, this pattern. Career contraction, reduced consumption, or postponed childbearing should not be interpreted as a culturally fixed preference. The narratives instead show how gender, household resources, and available substitutes narrowed or widened choices for particular participants.

### Implications for policy and practice

The findings suggest three policy directions, presented as implications rather than prescriptions derived directly from 23 interviews. First, financial support should be linked to an adequate supply of substitute care. Flexible allowances or service vouchers could help high-intensity, lower-income households purchase accredited childcare, home-based eldercare, or temporary respite, but only where services are available and trusted. Linking purchasing power to service supply also reduces the risk that cash support cannot be converted into actual relief from care. Evidence from China’s long-term care insurance pilots indicates that greater access to formal care can reduce some informal-care burden and influence labor participation [33].

Second, time protection should cover both childcare and care for older adults. Managerial support, flexible schedules, and work adjustment are important resources for employed informal caregivers [34], while paid family and medical leave may benefit worker health and family wellbeing [35]. Caregiver-inclusive leave, reversible reductions in hours, and flexible or hybrid work where job tasks permit could protect employment continuity. Public or social-insurance financing may reduce employer resistance, but safeguards are needed to prevent caregiving responsibilities from reinforcing women’s employment penalties [36].

Third, communities need a coordination point capable of identifying gaps across both care domains. It could connect childcare, home-based eldercare, respite, psychological support, and emergency back-up care, with opening hours aligned to work, medical visits, and school transport. Systematic reviews provide relevant evidence for respite care [37] and navigation programs linking health and community services [38]. Universal, affordable, and reliable early childhood education and care remain an important foundation for reducing family inequalities [39].

### Limitations

Several limitations constrain interpretation. The online questionnaire used non-probability recruitment, and interviewees self-selected by leaving contact details. The sample may therefore overrepresent people with digital access who were willing to discuss family experiences. The 23 participants came only from Shanghai and Xi’an and were predominantly women; they cannot represent all dual caregivers in China or support prevalence, statistical significance, or causal claims. Interview mode coincided partly with collection phase and location and may have affected rapport or disclosure. Translation of Chinese narratives into English may also have reduced semantic nuance.

### Conclusion

Dual caregivers in Shanghai and Xi’an did not simply experience the addition of two care burdens. They continually negotiated among competing demands, secondary strains, and available resources. Family help, formal and market services, and self-regulation widened choices only when they matched the relevant task and time, producing four changeable strategy configurations and varied adjustments to work, further childbearing, and future planning. This model extends the stress process into a feedback-oriented account of resource allocation: short-term arrangements can sustain care while transferring costs to health, employment, or future capacity. The explanation is grounded in 23 interviews and requires examination across other regions, household structures, and stages of care.

## Data Availability

The interview transcripts contain sensitive family narratives, and participants did not consent to public release. De-identified excerpts may be considered by the author for a clearly specified research purpose, subject to the ethics approval, the scope of participant consent, and applicable data-protection requirements.
