# Supplementary material for: Stress and coping among dual caregivers in China: a grounded theory study of intergenerational caregiving
Source: Int J Public Health. 2026 Sep 23;71:1610111. doi: 10.3389/ijph.2026.1610111 (PMC13642225; doi:10.3389/ijph.2026.1610111)
Supplement: Supplementary file 1 [file Table1.docx]

**Supplementary Material**

Demographic Details of Interviewees

| **ID** | **Gender** | **Age** | **Region** | **Education Level** | **Health Condition** | **Employment Status** | **Number of Children Under Care** | **Number of Old adults Under Care** |
| --- | --- | --- | --- | --- | --- | --- | --- | --- |
| 1 | Female | 63 | Xi'an | Below Primary | Chronic illness, cervical spondylosis | No | 2 | 1 |
| 2 | Female | 72 | Xi'an | Below Primary | Stomach pain | No | 1 | 1 |
| 3 | Female | 60 | Xi'an | Below Bachelor's | Chronic illness | No | 2 | 1 |
| 4 | Female | 45 | Xi'an | Below Bachelor's | Healthy | Yes | 1 | 2 |
| 5 | Female | 35 | Xi'an | Above Bachelor's | Healthy | Yes | 1 | 2 |
| 6 | Male | 34 | Xi'an | Bachelor's | Hyperthyroidism | Yes | 1 | 1 |
| 7 | Female | 35 | Xi'an | Bachelor's | Healthy | Yes | 2 | 2 |
| 8 | Female | 32 | Xi'an | Vocational | Healthy | Yes | 2 | 2 |
| 9 | Female | 35 | Xi'an | Associate's | Healthy | Yes | 2 | 2 |
| 10 | Female | 34 | Xi'an | Associate's | Healthy | Yes | 1 | - |
| 11 | Male | 53 | Xi'an | Junior High | Physical disability | No | 1 | 1 |
| 12 | Female | 60 | Xi'an | Vocational | Chronic illness, back pain | No | 1 | 1 |
| 13 | Female | 59 | Xi'an | Junior High | Healthy | No | 2 | 1 |
| 14 | Male | 32 | Shanghai | Bachelor's | Healthy | Yes | 1 | 2 |
| 15 | Female | 41 | Xi'an | Bachelor's | Healthy | No | 1 | 2 |
| 16 | Female | 39 | Shanghai | Associate's | Healthy | Yes | 1 | 2 |
| 17 | Male | 35 | Shanghai | Bachelor's | Healthy | Yes | 1 | 2 |
| 18 | Female | 46 | Xi'an | Above Bachelor's | Healthy | Yes | 2 | 1 |
| 19 | Male | 32 | Shanghai | Bachelor's | Healthy | Yes | 1 | 1 |
| 20 | Female | 36 | Shanghai | Bachelor's | Healthy | Yes | 1 | 1 |
| 21 | Female | 55 | Shanghai | Associate's | Healthy | No | 1 | 1 |
| 22 | Male | 57 | Shanghai | Associate's | Hepatitis B | Yes | 1 | 1 |
| 23 | Female | 34 | Shanghai | Bachelor's | Healthy | Yes | 1 | 1 |
